# Cortical gyrification deficits in early-stage Parkinson's disease: the importance of bradykinesia

**DOI:** 10.1093/braincomms/fcag094

**Published:** 2026-03-31

**Authors:** Antonio Callén, Gemma Colomé, Christian Stephan-Otto, Christian Núñez

**Affiliations:** Neurology Department, Parc Sanitari Sant Joan de Déu, Sant Boi de Llobregat, Barcelona 08830, Spain; Institut de Recerca Sant Joan de Déu, Esplugues de Llobregat, Barcelona 08950, Spain; Neurology Department, Parc Sanitari Sant Joan de Déu, Sant Boi de Llobregat, Barcelona 08830, Spain; Institut de Recerca Sant Joan de Déu, Esplugues de Llobregat, Barcelona 08950, Spain; Consorcio de Investigación Biomédica en Red de Salud Mental (CIBERSAM), Madrid 28029, Spain; Pediatric Computational Imaging Group (PeCIC), Hospital Sant Joan de Déu, Esplugues de Llobregat, Barcelona 08950, Spain; Mental Health Research Group, Institut d’Investigació Biomèdica Sant Pau (IIB Sant Pau), Hospital de la Santa Creu i Sant Pau, Barcelona 08025, Spain; Fundació de Recerca Clínic Barcelona - Institut d'Investigacions Biomèdiques August Pi i Sunyer (IDIBAPS), Barcelona 08036, Spain

**Keywords:** cortical folding, neuroimaging, neuroanatomy, neurology, functional capacity

## Abstract

Timely, accurate diagnosis of Parkinson's disease is still challenging for clinicians. It is therefore crucial to identify novel biomarkers to better characterize the early stages of the disease. Here, we assessed cross-sectional brain structure differences between healthy control (HC) and Parkinson's disease participants. We also explored potential correlations between brain structure and distinctive Parkinson's disease clinical features. We analysed T_1_-weighted brain images from 381 Parkinson's disease patients, primarily in the early stages, and 139 HC participants obtained from the Parkinson's Progression Markers Initiative (PPMI) database. The image processing protocol included quantification of several brain structure parameters: grey matter volume (GMV), cortical thickness, gyrification index (GI), sulcal depth and surface ratio. Regarding clinical variables, we gathered the Schwab and England score (as a measure of functional capacity), along with four motor symptom scores (bradykinesia, tremor, rigidity and postural instability) derived from the Movement Disorder Society-Unified Parkinson's Disease Rating Scale (MDS-UPDRS). We found that the left parahippocampal and lingual gyri showed less gyrification in Parkinson's disease patients compared to HC participants. In Parkinson's disease patients, we also identified GI deficits associated with bradykinesia, the main cardinal motor sign, in right parietal (mainly the supramarginal gyrus), temporal and occipital regions. In addition, higher GI and GMV in the occipital cortex were associated with greater functional capacity in Parkinson's disease. In conclusion, the gyrification deficits observed in early-stage Parkinson's disease patients point to the potential value of cortical folding as a biomarker in Parkinson's disease. Our results indicate that GI deficits are closely associated with bradykinesia and impaired functional capacity, possibly reflecting connectivity issues and/or compensatory mechanisms.

## Teaser

Callén *et al*. report that reduced gyrification in the supramarginal gyrus is associated with more severe bradykinesia in Parkinson’s disease patients, whereas higher gyrification in occipital areas relates to greater functional capacity. These findings suggest that cortical folding is a promising marker for early-stage Parkinson’s disease.

## Graphical Abstract

**Graphical Abstract:**
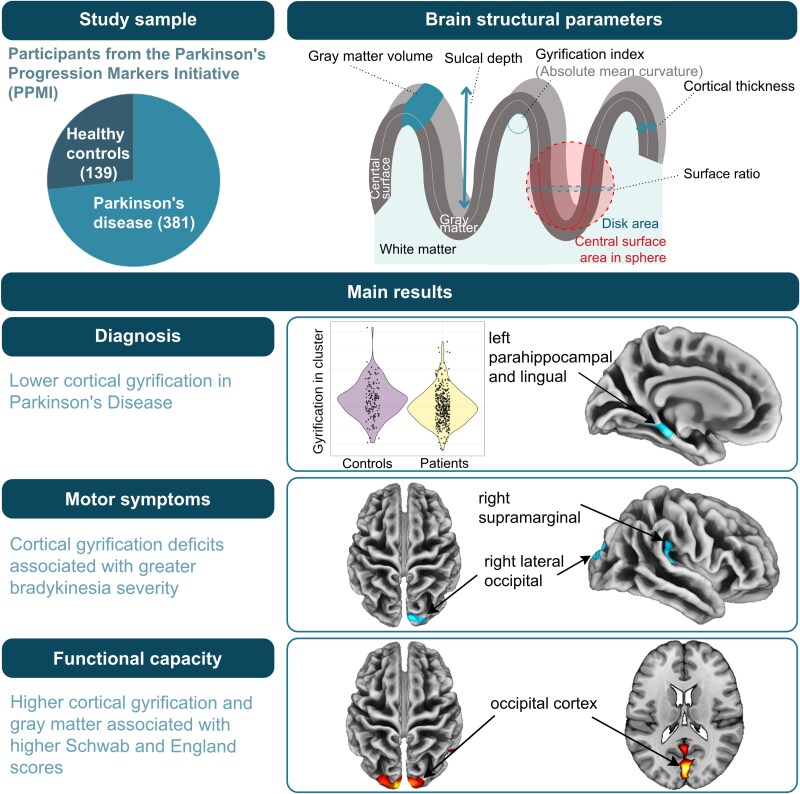

## Introduction

Parkinson's disease is a progressive neurodegenerative disorder characterized by both motor symptoms (such as bradykinesia, tremor, rigidity and postural instability) and non-motor symptoms (sleep disorders, cognitive impairment and affective disorders, among others).^1,2^ In addition to the characteristic degeneration of dopaminergic neurons in the substantia nigra, accumulation of alpha-synuclein inclusions extending to cortical areas has been reported in post-mortem analyses.^3^ This finding, together with the presence of non-motor symptoms, suggests that additional degeneration could occur in extranigral regions reaching the cortex.^4^ However, the mechanisms causing cortical pathology have not yet been described.

At present, accurate, objective diagnosis of the disease remains challenging. Misdiagnosis is a pervasive problem due to the use of subjective symptom rating scales and delayed diagnoses after the first symptoms are frequent. For this reason, early-stage diagnostic biomarkers such as genetic analysis and neuroimaging approaches are starting to be used as part of routine protocols in clinical practice, although with limited results.^5^

In recent years, neuroimaging techniques such as MRI have been used to study macroscopic structural changes in the brain of Parkinson's disease patients, which may help to improve our understanding of the underlying pathological patterns while also potentially providing *in vivo* biomarkers.^6^ In fact, early studies of brain integrity have revealed a reduction in grey matter volume (GMV) and cortical thickness (CT) in Parkinson's disease patients with respect to healthy individuals.^7,8^

In addition to the more conventional volumetric brain features, cortical folding parameters, including gyrification index (GI), sulcal depth (SD) and surface ratio (SR), may provide valuable information about the morphological complexity of the cortex in neurodegenerative disorders (see, for example, Núñez *et al*.^9^ and Zhang *et al*.^10^). Previous studies in Parkinson's disease have shown GI abnormalities associated with its diagnosis,^11^ duration of illness (DOI),^12,13^ cognitive impairment^14^ and motor phenotypes such as the akinetic-rigid subtype.^15^

In the present study, we compared gyrification and other brain integrity measures between Parkinson's disease patients and healthy control (HC) participants from the considerably large Parkinson’s Progression Markers Initiative (PPMI) study cohort.^16^ As suggested by the scarce but somewhat consistent existing literature, we expected to find brain structure deficits in Parkinson's disease patients. In a more exploratory manner, we tested for correlations between brain integrity measures and clinical features, namely, functional capacity (assessed with the Schwab and England scale) and various motor symptoms.

## Materials and methods

### Participants

We obtained data from a total of 520 participants from the openly available PPMI dataset (https://www.ppmi-info.org). Some inclusion criteria for Parkinson's disease patients at the Screening visit were age 30 years or older, diagnosis within the past 2 years and exhibiting at least two of the following symptoms: resting tremor, bradykinesia, rigidity or either asymmetric resting tremor or asymmetric bradykinesia. Detailed inclusion and exclusion criteria for patients and healthy participants can be consulted elsewhere [PPMI Clinical Protocol (002), ver. 30Jan2023_AM3.2]. The initial sample we considered was composed of 608 participants (445 Parkinson's disease and 163 HC), but since there was a very low percentage of Parkinson's disease or HC participants that did not identify as white (6%, *n* = 37), we excluded these from the analyses to avoid potential confounding issues. We additionally excluded 51 participants (9%) whose MRI data generated concerns (see ‘Neuroimaging data acquisition and processing’ section), thus resulting in a final study sample of 381 Parkinson's disease and 139 HC participants. Given the nature of the PPMI dataset, most of the patients included in our study were in the early stages of the disease [median DOI = 181 (IQR = 365) days; mean Movement Disorder Society-Unified Parkinson’s Disease Rating Scale (MDS-UPDRS) Part III total score = 21.2 (SD = 9.1); 98% of the patients were in the stages 1 or 2 of the Hoehn and Yahr scale].

### Neuroimaging data acquisition and processing

We collected high-resolution T_1_-weighted structural images from the baseline visit of the participants. MRI scans were acquired with either 1.5 T (*n* = 155) or 3 T scanners (*n* = 365); we did not find significant differences (*P* = 0.457) in the distribution of patients and controls in relation to the field strength of the scanner. The acquisition parameters for each scanner are shown in the Supplementary Methods. The PPMI imaging protocol may be found on the PPMI website (https://www.ppmi-info.org/study-design/research-documents-and-sops). We processed the structural images with CAT12 (v12.7, r1742),^17^ a toolbox for SPM12 (https://www.fil.ion.ucl.ac.uk/spm/software/spm12), which runs under MATLAB (Release 2020b, The MathWorks, Inc., Natick, MA, USA). A detailed description of the image processing procedure is available in the Supplementary Methods and in Gaser *et al*.^17^ In short, image processing consisted of segmentation of the grey matter, white matter and cerebrospinal fluid, as well as cortical surface reconstruction. This procedure yielded GMV maps for each participant, including both cortical and subcortical areas, which we smoothed with a Gaussian kernel of 8 mm full width at half maximum (FWHM). We employed these GMV maps for voxel-based morphometry (VBM) analyses. We also generated CT, GI, SD and SR maps for each participant. Briefly, CT is the distance between the inner (white matter/grey matter boundary) and outer (grey matter/cerebrospinal fluid boundary) surfaces. GI maps were estimated based on the curvature method of Luders *et al*.^18^ SD is defined as the Euclidean distance between the central surface (located between the inner and outer surfaces) and its convex hull, which is then square root transformed. Finally, the SR, also known as the Toro’s GI,^19^ is defined as the ratio between the central surface area contained in a sphere with a radius of 20 mm and the area of a disk of the same radius. We resampled these cortical surface maps to a high-resolution 164 k mesh and, following the recommendations from CAT12 developers, we smoothed them using a 12 mm FWHM filter for CT and a 20 mm FWHM filter for GI, SD and SR. We employed these maps to carry out surface-based morphometry (SBM) analyses. To precisely locate the results of the VBM and SBM analyses, we used the Neuromorphometrics (http://www.neuromorphometrics.com) and the Desikan-Killiany (DK40)^20^ atlases, respectively. We used the ‘Data quality’ tools available in CAT12, as well as the reports generated after image processing, to ensure that low-quality images or images that might have been processed inadequately were not included in our analyses. The specific criteria used to assess the quality of image processing are available in the Supplementary Methods. As mentioned above, we excluded 51 participants (9% of the total sample) after conducting the quality control procedure. Of those, 39 were Parkinson's disease patients and 12 were HC. The proportion of excluded Parkinson's disease and HC participants did not significantly differ from that of the included participants (*P* = 0.621). Similarly, we observed no differences in age, sex or education. Within the subgroup of patients, there were no statistically significant differences between included and excluded patients in any of the clinical variables assessed, nor in medication or DOI.

### Clinical data

We extracted scores for the modified Schwab and England Activities of Daily Living scale and the MDS-UPDRS, both available in the PPMI database. For the statistical analyses, we employed the Schwab and England score as a measure of functional capacity, and we computed four motor symptom scores based on the average of several MDS-UPDRS items: bradykinesia (items 3.4 to 3.8 and 3.14), tremor (items 3.15 to 3.18), rigidity (item 3.3) and postural instability (items 3.12 and 3.13). Since we did not observe brain structure differences between akinetic-rigid and tremor-dominant Parkinson's disease subtypes (see the ‘Preliminary analyses’ section in the Supplementary material), we included all patients in the analyses. Similarly, no brain structure differences were found between predominantly left- and right-affected patients (see ‘Preliminary analyses’); hence, for those items with multiple separate assessments (e.g. left and right sides of the body), we averaged across sides and did not distinguish between the more- and less-affected sides. To ensure that the scores were truly representative of patient status, in each analysis, we only included those participants showing a reasonable minimum time span (∼2 months) between administration of the clinical test and MRI acquisition. Since we did not know the exact day when the clinical tests were administered, this could result in a bias towards the inclusion of a larger number of participants who had the clinical test administered after the brain scan. To avoid this, we included participants who completed the clinical test up to 75 days before and up to 60 days after the MRI acquisition. Hence, we included a total of 372 patients in the Schwab and England analyses and 371 patients in the motor symptom analyses.

### Medication and duration of illness

We estimated the cumulative dose of Parkinson's disease medication (cLEDD) until MRI acquisition. This metric has previously been used in Parkinson's disease studies (see, for example, Scott *et al*.^21^). To do so, we multiplied the levodopa equivalent daily dose by the number of months of medication intake before the brain scan. We considered the following types of medication: levodopa, dopaminergic agonists, monoamine oxidase inhibitors and amantadine. We also obtained the date of diagnosis to estimate the DOI until MRI acquisition. Both cLEDD and DOI were employed as covariates to check the consistency of our analyses (see ‘Statistical analysis’ section).

### Statistical analysis

First, we compared sociodemographic and clinical data between Parkinson's disease patients and HC participants by means of *t*-tests and *χ*² tests, as appropriate. Next, aiming to identify potential brain structure differences between Parkinson's disease patients and HC participants, we performed whole-cortex ANCOVA VBM and SBM analyses of the GMV and cortical surface maps, respectively, with diagnosis (Parkinson's disease or HC) as the independent variable and age, sex and education level as covariates. Total intracranial volume was included as an additional covariate in VBM analyses. To explore potential associations between clinical data and brain structure in Parkinson's disease patients, we carried out additional whole-cortex multiple regression VBM and SBM analyses of the GMV and cortical surface maps. In this case, in each analysis, we employed one of the clinical scores, namely, Schwab and England, bradykinesia, tremor, rigidity and postural instability, as the independent variable, with age, sex, education level and total intracranial volume (the latter only in VBM analyses) as covariates. In order to test the robustness of our results, we repeated the latter analyses using cLEDD, DOI and field strength, one at a time, as additional covariates. Finally, we also explored the effects of cLEDD and DOI on brain structure. In all the VBM and SBM analyses, we used a voxel/vertex-level cluster-defining threshold of *P* < 0.001 and a family-wise error–corrected cluster-level threshold of *P* < 0.05. In the VBM analyses, we also used a GMV absolute masking threshold of 0.2. All these analyses used openly available data from PPMI.

### Ethical statement

The PPMI study was conducted in accordance with the Declaration of Helsinki, the Good Clinical Practice guidelines, and any applicable national and local regulations, after approval of the local Ethics Committees of the participating sites. All the participants signed an informed consent agreement.

## Results

### Sociodemographic and clinical information

Sociodemographic and clinical information of the participants are detailed in Table 1. There were no statistically significant differences between the Parkinson's disease and HC groups for any of the sociodemographic variables (i.e. age, sex and education).

**Table 1:** Sociodemographic and clinical data comparison between healthy controls and Parkinson’s disease patients

Variable	Parkinson's disease [mean ± SD or median (IQR), *n* = 381]	Healthy controls [mean ± SD or median (IQR), *n* = 139]	Differences
Age (years)	62.1 ± 9.4	61.0 ± 11.0	*P* = 0.259^a^
Age range (years)	33.6–83.0	30.6–81.9	
Sex (M/F)	235/146	86/53	*P* = 0.968^b^
Education (years)	15.8 ± 3.2	16.2 ± 2.9	*P* = 0.174^a^
Education range (years)	5–26	9–24	
Medication (yes/no)	81/300		
Medication range (mg)^c^	0–76 700		
Duration of illness (days)^d^	181 (365)		
Duration of illness range (days)^d^	13–2869		
Scanner field strength (3 T/1.5 T)	264/117	101/38	*P* = 0.457^b^
Hoehn and Yahr stage (1/2/3)	144/230/7		
MDS-UPDRS Part III total score	21.2 ± 9.1		
Schwab and England	92.9 ± 6.3		
Bradykinesia	0.82 (0.73)	0 (0)	*P* < 0.001^a^
Tremor	0.40 (0.40)	0 (0)	*P* < 0.001^a^
Rigidity	0.60 (0.80)	0 (0)	*P* < 0.001^a^
Postural instability	0.50 (0.50)	0 (0)	*P* < 0.001^a^
Geriatric Depression Scale	2 (2)	1 (1)	*P* < 0.001^a^
Montreal Cognitive Assessment	27 (3)	28 (2)	*P* < 0.001^a^

^a^Probability value derived from a *t*-test.

^b^Probability value derived from a *χ*² test.

^c^Cumulative dose of Parkinson's disease medication (levodopa equivalents) until MRI acquisition.

^d^Duration of illness from date of diagnosis until MRI acquisition.

### Brain structure differences between groups

**Figure 1:**
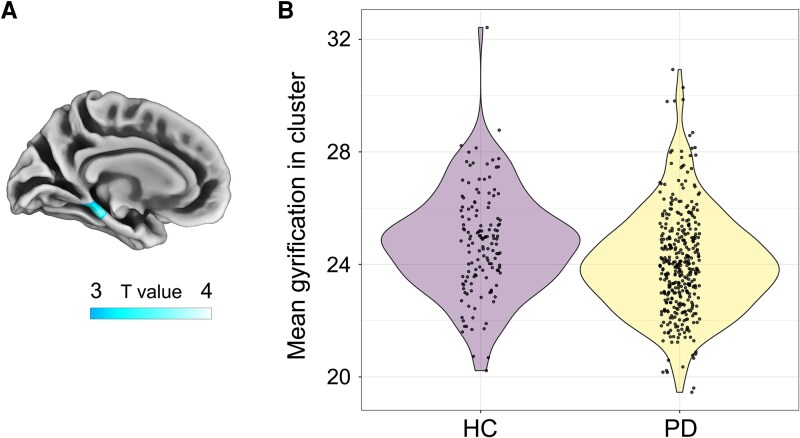
**Gyrification deficits in Parkinson's disease patients.** (**A**) Medial view of the left hemisphere of the brain showing the results from the ANCOVA voxel- and surface-based morphometry analyses. We found hypogyrification in Parkinson's disease (PD, *n* = 381) patients, compared to healthy controls (HC, *n* = 139), at the parahippocampal and lingual gyri (*P*_cluster_ = 0.035; *T*_peak_ value = 3.7). The colour bar represents *T* values. (**B**) Violin plot depicting mean gyrification differences in this cluster between HC and Parkinson's disease patients. Dots represent individual raw values.

The Parkinson's disease group presented a GI deficit in the left parahippocampal (93% of the hypogyrified region) and lingual (7%) gyri (cluster size = 685; *P*_cluster_ = 0.035; *T*_peak_ value = 3.7) (Figure 1). The HC group did not show GI deficits in any region when compared to Parkinson's disease. No statistical differences between groups were found for any other brain parameter.

### Association between brain structure and clinical assessment

We tested for potential correlations between the main clinical characteristics of Parkinson's disease and brain parameters (GMV, CT, GI, SD and SR). Only bradykinesia and functional capacity showed statistically significant correlations with brain structure measures. The rest of the clinical factors either did not present any statistically significant effects (tremor and rigidity), or else the effects did not survive the inclusion of cLEDD as a potentially confounding variable (postural instability).

**Figure 2:**
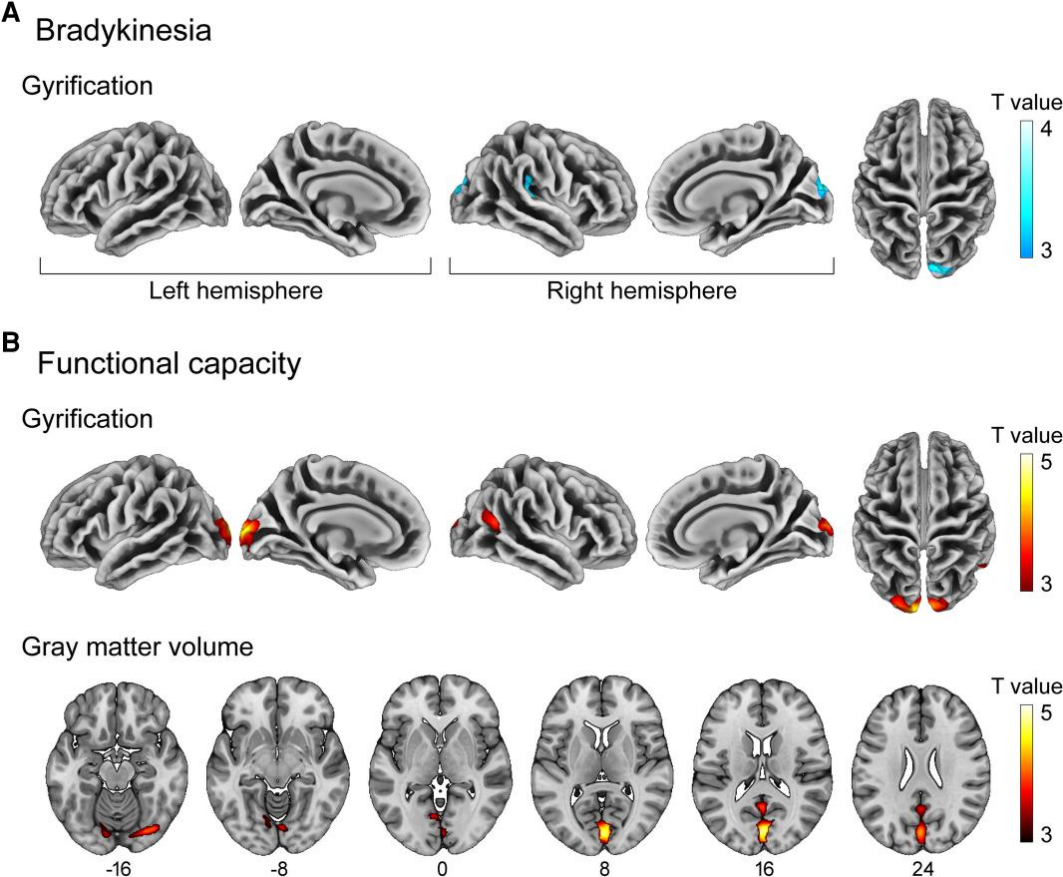
**The degree of gyrification is associated with bradykinesia and functional capacity.** Brain regions where brain structure parameters correlated with clinical characteristics in multiple regression voxel- and surface-based morphometry analyses. (**A**) Cortical gyrification deficits associated with bradykinesia in Parkinson's disease patients. (**B**) Higher GMV and cortical gyrification associated with the degree of functional capacity measured by means of the Schwab and England scale. Colour bars represent T values, with blue–white indicating a negative correlation and red–yellow a positive one. The numbers below axial slices denote the Z coordinates in the MNI anatomical space. See the scatter plots for these associations in Fig. 3 and the clusters listed in Table 2.

**Figure 3:**
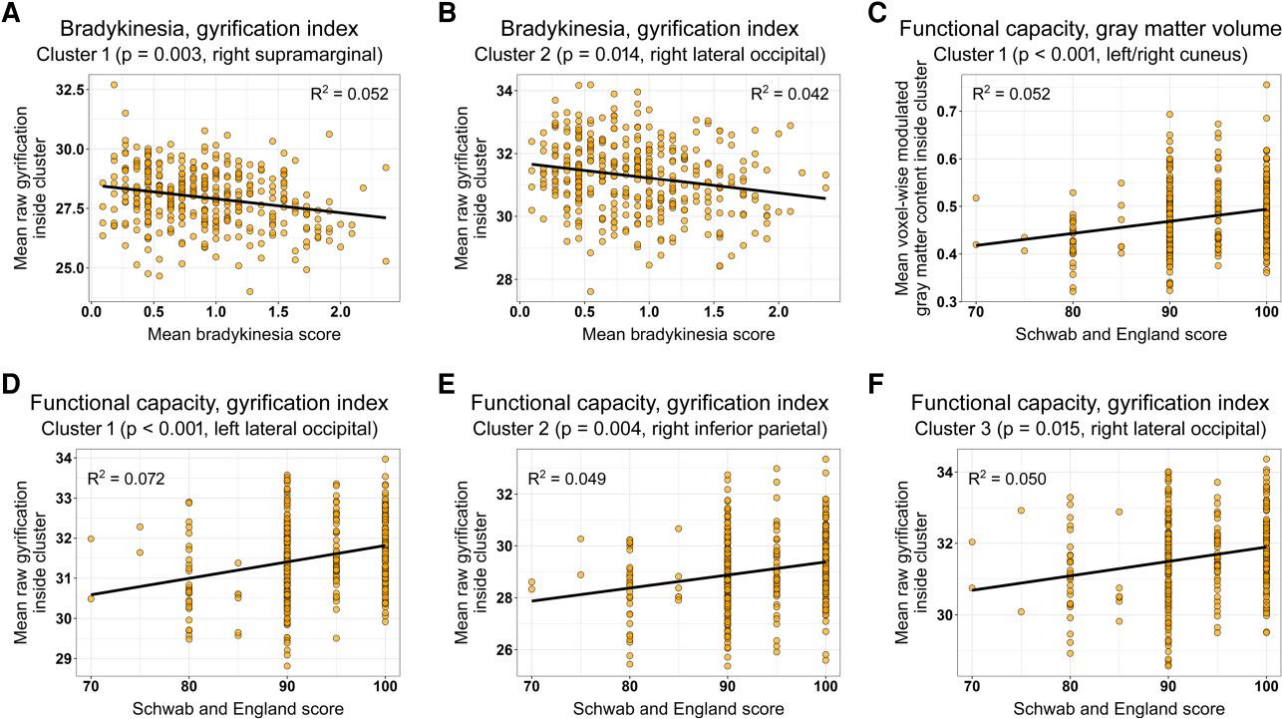
**Scatter plots of brain structure associations with bradykinesia and functional capacity.** Scatter plots for each of the significant clusters arising from the multiple regression voxel- and surface-based morphometry analyses of bradykinesia (**A** and **B**) and functional capacity (**C–F**; clusters depicted in Fig. 2 and listed in Table 2). Data points represent individual Parkinson's disease patients, showing bradykinesia or functional capacity scores plotted against mean gyrification or grey matter values.

In the case of bradykinesia (MDS-UPDRS items 3.4 to 3.8 and 3.14), we found a negative association with cortical gyrification. Specifically, higher bradykinesia scores were associated with decreased GI in two regions of the right hemisphere: one involving the supramarginal, superior temporal and transverse temporal gyri (cluster size = 1129; *P*_cluster_ = 0.003; *T*_peak_ = 3.8) and the other extending to the lateral occipital and superior parietal gyri, as well as the cuneus (cluster size = 861; *P*_cluster_ = 0.014; *T*_peak_ = 3.7). Figures 2A and 3 show the resulting clusters and the association scatter plots; Table 2 provides a detailed description of these clusters.

**Table 2:** Description of the clusters showing structural parameter associations with bradykinesia and functional capacity in Parkinson’s disease patients

Cluster size (*P* value)	Peak *T* value (MNI coordinates)	Region (cluster % within region)
Bradykinesia		
Gyrification index		
1129 (0.003)	3.8 (49, −20, 9)	Right supramarginal (70%)
		Right superior temporal (22%)
		Right transverse temporal (7%)
861 (0.014)	3.7 (14, −97, 20)	Right lateral occipital (45%)
		Right cuneus (35%)
		Right superior parietal (20%)
Functional capacity		
Grey matter volume		
3767 (< 0.001)	4.9 (0, −81, 11)	Left cuneus (17%)
		Right cuneus (16%)
		Left lingual gyrus (15%)
		Right occipital fusiform gyrus (9%)
		Right calcarine cortex (8%)
		Right precuneus (7%)
		Right lingual gyrus (7%)
		Left precuneus (7%)
Gyrification index		
2076 (< 0.001)	4.8 (−6, −97, 5)	Left lateral occipital (71%)
		Left cuneus (14%)
		Left pericalcarine (13%)
1083 (0.004)	3.9 (45, −58, 14)	Right inferior parietal (87%)
		Right middle temporal (13%)
831 (0.015)	4.2 (10, −100, 11)	Right lateral occipital (67%)
		Right cuneus (30%)

See Fig. 2 for a depiction of these clusters.

Regarding functional capacity, a statistically significant positive correlation was found between the Schwab and England score and GI and GMV mainly in the occipital cortex (Figs. 2B and 3 and Table 2). A similar association was observed between functional capacity and CT in the occipital lobe, as well as with SD in the right isthmus cingulate and right lingual regions, although these associations did not survive the inclusion of field strength and DOI, respectively, as potential confounders.

### Effects of medication and duration of illness on brain structure

Since cLEDD and DOI were included as potentially confounding variables, we explored their effects on brain structure. Our analyses revealed significant associations between these variables and CT, GI and SD. cLEDD correlated with higher CT in the left rostral middle frontal gyrus and the left superior frontal gyrus. A positive correlation was also observed between cLEDD and GI in the right pars triangularis and right lateral orbitofrontal gyrus. Longer DOI correlated with higher CT in the left postcentral/paracentral and bilateral superior temporal gyri, as well as with higher GI in the right lateral orbitofrontal gyrus and right pars orbitalis. Conversely, negative correlations were observed for both cLEDD and DOI with SD, predominantly in the bilateral insular cortex. No significant associations arose with SR. See Supplementary Figure 1 and Supplementary Tables 1 and 2 for a detailed description of the clusters derived from these associations.

## Discussion

In the present study, we observed that Parkinson's disease patients present a gyrification deficit in the left parahippocampal and lingual gyri when compared to HC individuals. In contrast, no group differences were found for other brain parameters. Moreover, we found lower GI to be associated with more severe bradykinesia, a cardinal motor sign in Parkinson's disease. We also observed that better functional capacity was associated with larger GMV and GI. Overall, our results point to the potential of GI as a sensitive, reliable neuroimaging biomarker of Parkinson's disease.

The Parkinson's disease group showed a gyrification deficit in a single region mostly integrated within the parahippocampal gyrus, which has been associated with visuospatial and memory functions.^22^ This same region, among others, presented hypogyrification in Parkinson's disease patients in a study by Zhang *et al*.^11^ A 4-year follow-up study by the same group showed a decrease in GI in some of the regions described here,^13^ including gyri proximal to the parahippocampal. In a late-stage cohort, Mo *et al*.^23^ reported a cortical folding deficit associated with Parkinson's disease mainly in occipital regions. In another study, Sterling *et al*.^12^ observed an overall gyrification deficit in addition to region-specific differences, although these were not consistent with those reported in our analysis. Their results were only found in later stages of disease progression (>10 years), whereas no significant differences were found in the early-stage group, which was practically equivalent to our cohort in terms of DOI. This discrepancy could be explained by several factors, such as the smaller sample size used in their study, the size imbalance between our Parkinson's disease and HC groups and differences in image acquisition parameters and processing software. In contrast with our results, Li *et al*.^24^ found no significant differences in GI between Parkinson's disease and HC samples, which they attributed to their patients having a DOI < 5 years. We further assessed cortical morphology using SD and SR, but no significant differences were observed between Parkinson's disease and HC. This contrasts with Wang *et al*.,^25^ who reported reduced SD in Parkinson's disease patients across several regions. Other studies have identified SD differences in specific Parkinson's disease subgroups relative to HC; for example, Li *et al*.^26^ found increased SD in the right supramarginal gyrus in patients exhibiting the tremor-dominant subtype, whereas Zhang *et al*.^10^ reported decreased SD in the left superior temporal sulcus in patients with right-sided Parkinson's disease onset.

This is the first study to associate bradykinesia, the main cardinal motor sign in Parkinson's disease (for an in-depth discussion of this concept, see Bologna *et al*.^27^), with a gyrification deficit. The resulting hypogyrification pattern includes the right supramarginal and superior temporal gyri, as well as a region spanning the right lateral occipital gyrus and the cuneus. The involvement of the supramarginal gyrus, part of the somatosensory association cortex, could be related to the defective integration of sensory information associated with bradykinesia (see, for example, the review by Bologna *et al*.^28^). It has been suggested that the degree of gyrification in a given brain structure is associated with connectivity^29^ and synaptic density.^30^ Therefore, we hypothesize that the hypogyrification observed in the supramarginal gyrus reflects a loss of neuronal connections in this region, which would in turn hinder the integration of sensory information, giving rise to, or aggravating, bradykinesia symptoms. We may alternatively hypothesize that those patients with lower bradykinesia scores have benefited from successful brain compensatory mechanisms, allowing them to preserve function and connectivity. In fact, increased functional resting-state connectivity was reported between the right supramarginal gyrus and the striatum for Parkinson's disease patients in Hacker *et al*.,^31^ which they attributed to a compensatory mechanism against function loss. Other motor deficits present in Parkinson's disease have also been associated with hypogyrification in recent studies, although the reported regions were different from those we observed. For example, Tang *et al*.^15^ reported precentral and postcentral gyrus hypogyrification in the Parkinson's disease akinetic-rigid subtype, pointing to the possibility that cortical gyrification abnormalities in Parkinson's disease are motor phenotype-specific. Interestingly, Sterling *et al*.^12^ carried out a longitudinal analysis and observed gyrification loss in the supramarginal gyrus in their middle-stage group of Parkinson's disease patients. Another motor-related symptom, freezing of gait, has also been associated with hypogyrification of the parahippocampal gyrus and the entorhinal cortex.^32^ In our study, other characteristic clinical motor symptoms of Parkinson's disease were also analysed, such as tremor, postural instability and rigidity, yet none of them presented an association with the assessed brain structure parameters. Of note, the study by Dehghan and Sarbaz^33^ showed that fractal dimension, another measure of cortical complexity, was significantly reduced in the postural instability gait difficulty subtype compared with both tremor-dominant patients and HC. Thus, future studies assessing fractal dimension may provide new insights into the relationship between cortical measures and motor symptoms in Parkinson's disease.

We also probed the impact of functional capacity, quantified by the Schwab and England scale, on brain morphology. In this regard, we found that better functioning was associated with higher GI and GMV in occipital regions of the cortex. This result, along with our observations regarding bradykinesia and group differences, suggests that the morphology of the occipital cortex and nearby areas is a promising biomarker of Parkinson's disease. Atrophy of the occipital lobe has previously been associated with visuospatial and visuoperceptual impairments, which provides a potential explanation for the progression to dementia observed in a considerable percentage of Parkinson's disease patients.^34^

We also analysed the effects on brain structure of the two main confounding variables considered in this study, cLEDD and DOI. cLEDD was positively correlated with CT in the left frontal gyrus. The same association was found with GI in the right pars triangularis and right lateral orbitofrontal gyrus. A positive correlation between DOI and CT was found specifically in the left postcentral/precentral and bilateral superior temporal gyri as well as with GI in the right lateral orbitofrontal gyrus and right pars orbitalis. Interestingly, some of the significant regions arising from our cLEDD/DOI and CT correlation analyses, such as the precentral/postcentral and the superior frontal gyri, showed a negative association with GI in the study of Sterling *et al*.^12^ This is somewhat consistent with the notion that CT and GI show opposite trends.^35^ Even though Sterling *et al*.^12^ observed an overall gyrification decrease in association with medication and DOI, which would contradict our results, it must be noted that they did not assess the specific regions where we found the associations with GI. Moreover, they employed a sample with a more progressed disease in terms of DOI and used a different GI measure. Conversely, SD was negatively correlated with both cLEDD and DOI, predominantly in the bilateral insular cortex. To the best of our knowledge, only one study has previously assessed the relationship between SD and medication in Parkinson's disease and found no significant relationships.^36^ It must be noted, though, that their analysis was limited to the olfactory sulcus. Cortical alterations of the insula have already been reported in neurodegenerative disorders, including Parkinson's disease.^9,25^ The functional importance of the insula in Parkinson's disease, particularly in relation to non-motor symptoms and medication intake, has been further highlighted by Criaud *et al*.^37^ Altogether, our results are consistent with the interpretation that higher cLEDD and longer DOI may reflect treatment-related and compensatory cortical effects. This is in line with the study by Johansson *et al*.,^38^ showing that disease progression in Parkinson's disease is shaped by longitudinal changes in cortical compensation, particularly in premotor regions, independent of nigrostriatal degeneration. In any case, cLEDD and DOI should be taken into account in future studies on Parkinson's disease involving cortical measures. These variables could also aid in the interpretation of previous literature.

This study has several strengths. To begin with, we used the well-known PPMI database, which is also a considerably large sample. In addition, we used a whole-brain approach, together with a well-established methodology previously used in similar studies.^9^ Controlling the effect of potential confounds such as DOI and cLEDD added robustness to the results of this study. There are also some limitations which need to be considered. The use of large databases has some disadvantages, such as the potential effects derived from inter-centre variability, and the use of multiple MRI scanners. Given the small number of participants that were imaged in some of the scanners, we did not harmonize MRI data, which may have affected our results. This issue has also been noted in previous studies.^39^ To partially account for this, we considered field strength as a potential confounder. It is also important to note that this is a cross-sectional study, since we analysed only brain scans from the baseline visit. Therefore, we were unable to draw conclusions about the utility of GI as a progression biomarker in Parkinson's disease, which should be addressed by future studies employing longitudinal designs. In addition, since PPMI in particular includes mostly early-stage Parkinson's disease patients, late stages of the disease are underrepresented. Furthermore, the sample sizes of the Parkinson's disease and HC groups were imbalanced, which could have affected the results of our group analysis. However, the design of this analysis assumed unequal variances, making it more robust and reducing the risk of false positive. Finally, our sample included only participants who identified as white. Because evidence suggests differences across ethnic groups in the prevalence, clinical progression and treatment response of Parkinson's disease,^40,41^ as well as in brain morphology,^42^ the lack of ethnic diversity in our study restricts the generalizability of our findings to other populations.

Our results suggest that cortical folding is a promising biomarker of disease progression and severity and could even facilitate early diagnosis. Further research to explore GI abnormalities in the context of Parkinson's disease should be undertaken.

## Supplementary Material

fcag094_Supplementary_Data

## Data Availability

Data used in the preparation of this article were obtained on January 2022 from the Parkinson’s Progression Markers Initiative (PPMI) database (www.ppmi-info.org/access-data-specimens/download-data), RRID:SCR_006431. For up-to-date information on the study, visit www.ppmi-info.org.
