# Supplementary material for: Cortical gyrification deficits in early-stage Parkinson's disease: the importance of bradykinesia
Source: Brain Commun. 2026 Mar 31;8(2):fcag094. doi: 10.1093/braincomms/fcag094 (PMC13037470; doi:10.1093/braincomms/fcag094)
Supplement: fcag094_Supplementary_Data [file fcag094_supplementary_data.docx]

**Cortical gyrification deficits in early-stage Parkinson’s disease: the importance of bradykinesia**

Antonio Callén, Gemma Colomé, Christian Stephan-Otto, Christian Núñez

**Supplementary Material**

**Supplementary Methods**

**Scanner acquisition parameters**

The most common acquisition parameters employed in each of the scanners are shown below.

| **Scanner** | **Field strength** | **Repetition time (TR)** | **Echo time (TE)** | **Inversion time (TI)** | **Slice thickness** | **Flip angle** | **Voxel size** | **Matrix size** |
| --- | --- | --- | --- | --- | --- | --- | --- | --- |
| Philips Achieva 1.5 T | 1.5 T | 7.1 ms | 3.2 ms | - | 1 mm | 8º | 1 x 1 x 1 mm^3^ | 256 x 256 |
| Philips Achieva 3 T | 3 T | 6.9 ms | 3.2 ms | - | 1.2 mm | 8º | 0.9 x 0.9 x 1.2 mm^3^ | 288 x 288 |
| Siemens Biograph mMR | 3 T | 2300 ms | 3 ms | 900 ms | 1 mm | 9º | 1 x 1 x 1 mm^3^ | 256 x 240 |
| General Electrics Discovery MR750(w) | 3 T | 7.6 ms | 3.1 ms | 400-600 ms | 1.2 mm | 8-13º | 1 x 1 x 1.2 mm^3^ | 256 x 256 |
| Siemens Espree | 1.5 T | 1970 ms | 3.2 ms | 1100 ms | 1 mm | 15º | 1 x 1 x 1 mm^3^ | 256 x 256 |
| General Electrics Genesis Signa | 1.5 T | 13 ms | 4.2 ms | 400 ms | 1.4 mm | 15º | 0.9 x 0.9 x 1.4 mm^3^ | 256 x 256 |
| Philips Intera | 1.5 T | 8.6 ms | 4 ms | - | 1.2 mm | 8º | 0.9 x 0.9 x 1.2 mm^3^ | 256 x 256 |
| General Electrics Optima MR450w | 1.5 T | 8.1 ms | 2.7 ms | - | 2 mm | 20º | 0.4 x 0.4 x 2 mm^3^ | 512 x 512 |
| Siemens Prisma (fit) | 3 T | 2300 ms | 2.9 ms | 900 ms | 1 mm | 9º | 1 x 1 x 1 mm^3^ | 256 x 256 |
| General Electrics Signa Excite | 1.5 T | 8.3 ms | 4 ms | - | 1.5 mm | 15º | 0.9 x 0.9 x 1.5 mm^3^ | 256 x 256 |
| General Electrics Signa HDxt 1.5 T | 1.5 T | 8 ms | 2.6 ms | - | 2 mm | 15º | 0.4 x 0.4 x 2 mm^3^ | 512 x 512 |
| General Electrics Signa HDxt 3 T | 3 T | 9.5 ms | 3.1 ms | 450 ms | 1.2-1.4 mm | 13-15º | 1 x 1 x 1.2-1.4 mm^3^ | 256 x 256 |
| Siemens Skyra | 3 T | 2300 ms | 3 ms | 900 ms | 1.2 mm | 9º | 1.1 x 1.1 x 1.2 mm^3^ | 256 x 240 |
| Siemens Symphony | 1.5 T | 1980-2400 ms | 3.7-3.9 ms | 1000-1100 ms | 1-3-2 mm | 8-15º | 1.3 x 1.3 x 1.3 mm^3^ – 0.5 x 0.5 x 2 mm^3^ | 192 x 192 – 512 x 512 |
| Siemens TrioTim | 3 T | 2300 ms | 3 ms | 900 ms | 1 mm | 9º | 1 x 1 x 1 mm^3^ | 256 x 240 |
| Siemens Verio | 3 T | 2300 ms | 3 ms | 900 ms | 1-1.2 mm | 9º | 1 x 1 x 1 mm^3^ – 1.1 x 1.1 x 1.2 mm^3^ | 256 x 240 |

**Voxel-based and surface-based processing**

The voxel-based processing performed by CAT12 is divided into two steps: tissue segmentation and spatial registration. The tissue segmentation procedure begins with the application of a denoising filter and the standard SPM unified segmentation. This is followed by CAT12’s tissue segmentation, which involves brain parcellation (left and right hemispheres, subcortical areas, ventricles, and cerebellum) and detection of local white matter hyperintensities, local intensity transformations to reduce the effects of higher gray matter intensities in several brain regions, and the application of an adaptive maximum a posteriori (AMAP) segmentation, which includes partial volume estimation. The individual tissue segments are then registered to the MNI space by means of a geodesic shooting registration algorithm. This procedure yields voxel-wise modulated gray matter volume maps, which are then spatially smoothed (in our case, using a Gaussian kernel of 8 mm full width at half maximum [FWHM]) and employed in voxel-based morphometry (VBM) analysis.

The surface-based processing relies on a projection-based thickness (PBT) method that estimates the initial cortical thickness and initial central surface, handling partial volume information, sulcal blurring, and sulcal asymmetries. Topological defects are then repaired with spherical harmonics. After a surface refinement, the final central, pial, and white surface meshes are generated. The initial cortical thickness estimate is refined using the pial and white matter surfaces. Cortical folding metrics can be calculated using the final central surface. Finally, the individual central surfaces are registered to the FreeSurfer “FsAverage” template. This procedure results in the generation of vertex-wise surface maps, which are then resampled and smoothed. In our case, we used a 12 mm FWHM filter for the smoothing of cortical thickness maps, and 20 mm FWHM filters for the smoothing of cortical folding maps (i.e., gyrification index, sulcal depth, and surface ratio). A full description of the voxel-based and surface-based processing procedures can be found in Gaser et al.^1^

**Specific criteria for the assessment of image processing quality**

Participants who met at least one of the specific criteria listed below, obtained from CAT12 reports after image processing, and/or were identified by the “Check sample homogeneity” tool (run for both VBM and surface data), were flagged as potentially defective. These participants were excluded after careful visual inspection of their brain scans and segmentation outputs if any of these were of poor quality and/or showed significant artifacts. There were no significant differences in any of the assessed quality variables between the Parkinson’s disease and healthy control participants that were included in the final sample.

Specific quality criteria:

- Noise rating >= 3
- Bias rating >= 2.75
- Image quality rating (IQR) >= 2.75
- Euler number >= 70
- Defect number >= 50

**Preliminary Analyses**

We conducted preliminary analyses considering Parkinson’s disease (PD) motor subtypes and lateralization of motor symptoms. First, we stratified patients into tremor-dominant (TD, n = 106) and akinetic-rigid (AR, n = 240) motor subtypes. To do this, we calculated TD and AR scores using the tremor and rigidity items, respectively, from the MDS-UPDRS, and computed the TD/AR ratio. The specific items and cutoff scores employed can be found in Adams et al.^2^ Patients with a mixed subtype (n = 24) were excluded from this analysis. Second, we classified patients based on their most affected side. We summed the left-sided (3.3c, 3.3e, 3.4b, 3.5b, 3.6b, 3.7b, 3.8b, 3.15b, 3.16b, 3.17b, 3.17d) and right-sided (3.3b, 3.3d, 3.4a, 3.5a, 3.6a, 3.7a, 3.8a, 3.15a, 3.16a, 3.17a, 3.17c) MDS-UPDRS items for tremor, bradykinesia, and rigidity symptoms, and computed the left/right ratio. Patients with a ratio > 1 were considered predominantly left-affected (n = 168), while those with a ratio < 1 were considered predominantly right-affected (n = 195). Patients without lateralization of motor symptoms (ratio = 1, n = 8) were excluded from this analysis.

We performed whole-cortex ANCOVA VBM and surface-based morphometry (SBM) analyses of the gray matter volume and cortical surface maps, respectively, with motor subtype (TD or AR) or lateralization (predominantly left- or right-affected) as the independent variable, and age, sex, and education level as covariates. Total intracranial volume was included as an additional covariate in VBM analyses. We used a voxel/vertex-level cluster-defining threshold of p < 0.001 and a family-wise error (FWE)-corrected cluster-level threshold of p < 0.05. In the VBM analyses, we also used a gray matter volume absolute masking threshold of 0.2.

None of the analyses yielded significant differences between PD motor subtypes or between predominantly left- and right-affected patients for any of the parameters analyzed (gray matter volume, cortical thickness, gyrification index, sulcal depth, and surface ratio).

**Supplementary Results**

**Supplementary Figure 1. Brain structure associations with medication and duration of illness.**


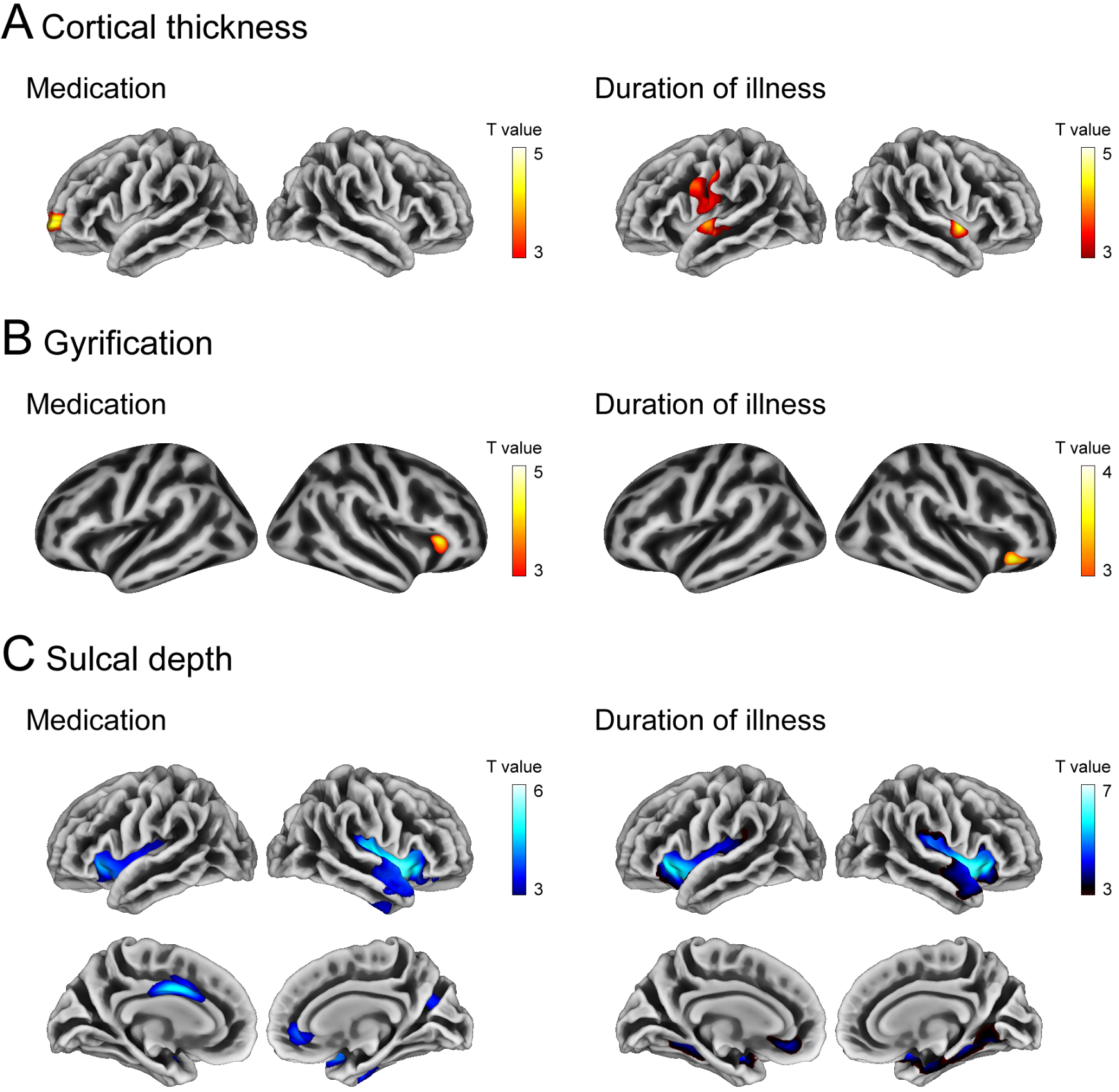
Brain regions showing **A.** higher cortical thickness, **B.** higher gyrification, and **C.** lower sulcal depth associated with higher medication and longer duration of illness in Parkinson’s disease patients (n = 381). Results were obtained using multiple regression voxel- and surface-based morphometry analyses. Gyrification results are displayed on inflated cortical surfaces for visualization purposes. Color bars represent T values.

**Supplementary Table 1.** Description of the clusters for which structural measures correlate with medication in Parkinson’s disease patients.

| **Cluster size**  **(p value)** | **Peak T value**  **(MNI coordinates)** | **Region**  **(cluster % within region)** |
| --- | --- | --- |
|  | | |
| ***Cumulated levodopa equivalent daily dose (cLEDD)*** | | |
| **Cortical thickness** | | |
| 654 (0.013) | 4.2 (-21, 63, -5) | Left rostral middle frontal (67%) |
|  |  | Left superior frontal (25%) |
|  |  | Left frontal pole (8%) |
| **Gyrification index** | | |
| 741 (0.022) | 4.1 (34, 29, 6) | Right pars triangularis (87%) |
|  |  | Right lateral orbitofrontal (13%) |
| **Sulcal depth** | | |
| 12,905 (< 0.001) | 5.0 (38, 5, -15) | Right insula (39%) |
|  |  | Right superior temporal (27%) |
|  |  | Right lateral orbitofrontal (11%) |
|  |  | Right transverse temporal (5%) |
|  |  | Right supramarginal (5%) |
| 6,063 (< 0.001) | 4.2 (-30, 18, -8) | Left insula (67%) |
|  |  | Left lateral orbitofrontal (16%) |
|  |  | Left superior temporal (9%) |
|  |  | Left transverse temporal (5%) |
| 2,532 (< 0.001) | 4.6 (-2, -2, 33) | Left posterior cingulate (50%) |
|  |  | Left caudal anterior cingulate (31%) |
|  |  | Left superior frontal (19%) |
| 901 (0.031) | 3.9 (9, 39, -7) | Right medial orbitofrontal (51%) |
|  |  | Right rostral anterior cingulate (33%) |
|  |  | Right superior frontal (16%) |
| 851 (0.033) | 3.7 (43, -16, -24) | Right inferior temporal (99%) |
| 798 (0.037) | 3.9 (21, -65, 27) | Right precuneus (72%) |
|  |  | Right cuneus (28%) |

**Supplementary Table 2.** Description of the clusters for which structural measures correlate with duration of illness in Parkinson’s disease patients.

| **Cluster size**  **(p value)** | **Peak T value**  **(MNI coordinates)** | **Region**  **(cluster % within region)** |
| --- | --- | --- |
| ***Duration of illness (DOI)*** | | |
| **Cortical thickness** | | |
| 2,472 (< 0.001) | 4.4 (-60, -5, 33) | Left postcentral (55%) |
|  |  | Left precentral (45%) |
| 949 (0.001) | 4.2 (-58, -2, -8) | Left superior temporal (100%) |
| 522 (0.036) | 4.7 (58, 6, -10) | Right superior temporal (100%) |
| **Gyrification index** | | |
| 673 (0.033) | 3.8 (34, 22, -13) | Right lateral orbitofrontal (91%) |
|  |  | Right pars orbitalis (9%) |
| **Sulcal depth** | | |
| 20,069 (< 0.001) | 6.1 (41, -0, -18) | Right insula (25%) |
|  |  | Right superior temporal (17%) |
|  |  | Right lingual (9%) |
|  |  | Right supramarginal (7%) |
|  |  | Right lateral orbitofrontal (7%) |
|  |  | Right postcentral (6%) |
|  |  | Right parahippocampal (5%) |
|  |  | Right fusiform (5%) |
| 13,940 (< 0.001) | 5.6 (-28, 19, -8) | Left insula (37%) |
|  |  | Left superior temporal (17%) |
|  |  | Left lateral orbitofrontal (12%) |
|  |  | Left transverse temporal (7%) |
|  |  | Left postcentral (6%) |
|  |  | Left pars triangularis (5%) |
|  |  | Left precentral (5%) |
| 1,387 (0.001) | 3.9 (-31, -46, -9) | Left fusiform (60%) |
|  |  | Left lingual (28%) |
|  |  | Left parahippocampal (13%) |
| 799 (0.020) | 4.0 (-7, 31, -14) | Left medial orbitofrontal (65%) |
|  |  | Left rostral anterior cingulate (35%) |

**Supplementary references**

1. Gaser C, Dahnke R, Thompson PM, Kurth F, Luders E, The Alzheimer’s Disease Neuroimaging Initiative. CAT: a computational anatomy toolbox for the analysis of structural MRI data. *Gigascience*. 2024;13:giae049.

2. Adams C, Suescun J, Haque A, et al. Updated Parkinson’s disease motor subtypes classification and correlation to cerebrospinal homovanillic acid and 5-hydroxyindoleacetic acid levels. *Clin Park Relat Disord*. 2023;8(100187):100187.
